# Global analysis of uncapped mRNA changes under drought stress and microRNA-dependent endonucleolytic cleavages in foxtail millet

**DOI:** 10.1186/s12870-015-0632-0

**Published:** 2015-10-06

**Authors:** Fei Yi, Jian Chen, Jingjuan Yu

**Affiliations:** State Key Laboratory of Agrobiotechnology, College of Biological Sciences, China Agricultural University, Beijing, 100193 China; State Key Laboratory of Agrobiotechnology, College of Agriculture and Biotechnology, China Agricultural University, Beijing, 100193 China

**Keywords:** Uncapped mRNA, Drought stress, PARE, Foxtail millet, miRNA target, miRNA precursor, C_4_

## Abstract

**Background:**

mRNA degradation plays an important role in the determination of mRNA abundance and can quickly regulate gene expression. The production of uncapped mRNAs, an important mechanism of mRNA degradation, can be initiated by decapping enzymes, endonucleases or small RNAs such as microRNAs (miRNAs). Little is known, however, about the role of uncapped mRNAs in plants under environmental stress.

**Results:**

Using a novel approach called parallel analysis of RNA ends (PARE), we performed a global study of uncapped mRNAs under drought stress in foxtail millet (*Setaria italica* [L.] P. Beauv.). When both gene degradation (PARE) and gene transcription (RNA-sequencing) data were considered, four types of mRNA decay patterns were identified under drought stress. In addition, 385 miRNA–target interactions were identified in the PARE data using PAREsnip. The PARE analysis also suggested that two miRNA hairpin processing mechanisms—loop-last and loop-first processing—operate in foxtail millet, with both miR319 and miR156 gene families undergoing precise processing via the unusual loop-first mechanism. Finally, we found 11 C_4_ photosynthesis-related enzymes encoded by drought-responsive genes.

**Conclusions:**

We performed a global analysis of mRNA degradation under drought stress and uncovered diverse drought-response mechanisms in foxtail millet. This information will deepen our understanding of mRNA expression under stressful environmental conditions in gramineous plants. In addition, PARE analysis identified many miRNA targets and revealed miRNA-precursor processing modes in foxtail millet.

**Electronic supplementary material:**

The online version of this article (doi:10.1186/s12870-015-0632-0) contains supplementary material, which is available to authorized users.

## Background

Transcript abundance is modulated by transcript synthesis and degradation rates. In recent years, genome-wide profiling methods, such as RNA-sequencing (RNA-seq) [1] and gene-chip analysis [2], have been used to study mRNA expression and to identify genes expressed in specific tissues and developmental processes [3] and in response to environmental stimuli [4]. Such studies are generally designed to capture aspects of steady-state mRNA abundance. In addition to transcriptional regulation, however, an understanding of gene expression networks obviously requires data on mRNA degradation and other patterns of mRNA expression regulation. Recent research has indicated that proper mRNA degradation is a component of cellular homeostasis maintenance and contributes to the precise adjustment of gene expression levels in response to various extracellular stimuli [5]. Several highly conserved pathways for mRNA degradation exist in eukaryotes. One such pathway proceeds in the 3′ to 5′ direction, with mRNA decay beginning with deadenylation catalyzed by mRNA deadenylases [6, 7]. Another mRNA degradation pathway, from 5′ to 3′, is often initiated with cleavage of the monomethyl guanosine (m^7^G) by the Dcp2 decapping enzyme [8, 9]. Dcp2 mainly cleaves m^2,2,7^G-capped RNA and m^7^G-capped RNA, while unmethylated capped RNA is a poor substrate [10]. In addition, some internal cleavages, such as those involving the RNA-induced silencing complex (RISC) directed by microRNAs (miRNAs) or small interfering RNAs, can initiate mRNA degradation [11, 12]. miRNA-mediated degradation mainly occurs in the miRNA:mRNA pairing binding region [13]. Similar mRNA decay mechanisms have been reported in plants, with some studies indicating that mRNA degradation is important for the control of gene expression during growth, development and many physiological transitions [14–16]. The most recent study on this topic revealed that RNA decay pathways function mainly via protecting transgenes and endogenous genes from inappropriate posttranscriptional gene silencing to regulate gene expression, and act as safeguards of plant development in Arabidopsis [17].

miRNAs are ~21-nucleotide non-coding RNAs that regulate gene expression by base-pairing to their targets, resulting in gene degradation or translational inhibition in most eukaryotes. In plants, the miRNA is almost completely complementary to its target gene and mediates cleavage of the target at the center of the paired region [18–20]. The high-throughput method used to discover miRNA targets in plants, which is based on computational prediction using a set of pre-defined rules [21, 22], leads to a large number of false positives. A more reliable, experimentally based approach involves the use of gene-specific 5′ rapid amplification of cDNA ends (RACE) to validate miRNA-target pairs [23]. Using this method, however, every single predicted target must be independently verified, which is labor-intensive, time-consuming and expensive. Although many miRNA-target pairs have been predicted, only a small fraction has been experimentally confirmed. One encouraging development is parallel analysis of RNA ends (PARE), a novel approach for identification of miRNA targets that is both high-throughput and reliable [24]. Using this method, large-scale miRNA–target pairs have been validated in species such as Arabidopsis [25, 26], rice [27, 28] and grapevine [29].

Most miRNA/miRNA* duplexes released from typical miRNA precursor stem-loops undergo two cycles of cleavage by endonucleases: one at the loop-distal position and the other at the loop-proximal position [30]. In animals, the two steps are spatially separated and completed by two different enzymes; specifically, duplexes are first cleaved by Drosha at the loop-distal position in the nucleus and then by Dicer at the loop-proximal position in the cytoplasm [31, 32]. In plants, the two sequential cleavages are both carried out by a Dicer-like (DCL) enzyme (DCL1) in the nucleus but the DCL1 sequential cleavage site is still poorly understood. Recent research has shown that PARE data can be used to probe patterns of miRNA hairpin processing in plants [33, 34].

Foxtail millet (*Setaria italica* [L.] P. Beauv.; Poaceae) is an important grass crop species widely planted in China. The genome of the foxtail millet cultivar Yugu1 has been sequenced recently [35]. Because of its trivial size and short life cycle, foxtail millet is an ideal model species for Panicoideae crops and C_4_ photosynthetic species [36–38]. Over the long period of its improvement and domestication, foxtail millet has gradually adapted to semiarid and arid climates. Because of its excellent drought tolerance and water-use efficiency, foxtail millet is an ideal material to investigate the mechanisms of drought tolerance in plants. To deepen our understanding of mRNA degradation and stress response mechanisms in plants, we therefore used the PARE deep-sequencing approach in this study to analyze uncapped mRNA transcripts under drought stress conditions in foxtail millet. PARE was also used to identify potential targets for miRNA-directed cleavage and to reveal multiple novel examples of miRNA precursor processing in foxtail millet.

## Results

### Overview of PARE-seq data in foxtail millet

To characterize mRNA degradation changes during the drought stress response, we profiled uncapped transcripts using PARE-seq (see Additional file 1 for details) in 14-day-old foxtail millet seedlings. PARE libraries were prepared from seedlings subjected to polyethylene glycol (PEG)-simulated drought (Dd) and control (Dc) conditions, with two independent biological replicates for each group. We generated approximately 39 million and 45 million 50-bp reads from the control and drought-treated seedlings, respectively (Additional file 2). After removing repeats/transposons [39] and known non-coding RNA (rRNA, tRNA, small nuclear RNA and small nucleolar RNA) [40] sequences as described in the Methods, the raw reads were mapped to the Yugu1 reference genome [35] using bowtie. The mapped reads, which accounted for about 68.06–76.35 % of the total reads from different PARE-seq libraries, were used to measure gene degradation levels calculated as reads per million (RPM) (see Methods). Because comparisons of biological replicates showed that their expression values were highly correlated (average *R*^*2*^ = 0.97; Additional file 3), we took the average RPM of the replicates as the degradation level.

**Fig. 1:**
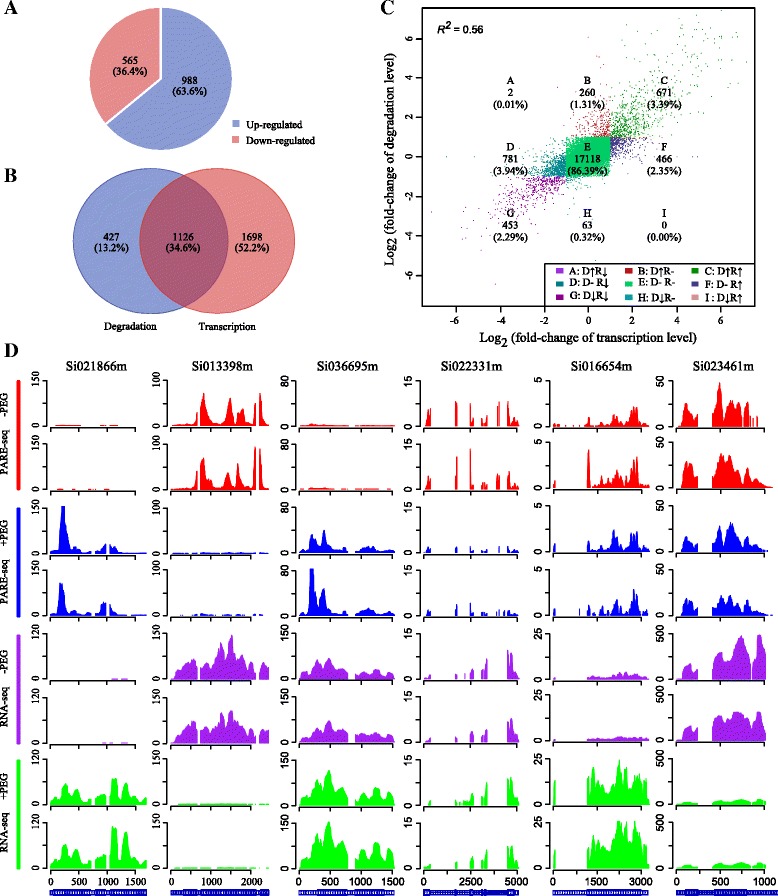
Drought stress-responsive genes at the degradation and transcription levels. **a** Number of genes showing significant degradation level changes after drought treatment. **b** Venn diagram showing significantly changed genes at the degradation and transcription levels under drought treatment. **c** Correlation between fold-change values at the degradation and transcription levels after drought stress. Correlation values (*R* ^*2*^) are Pearson’s product–moment correlation coefficients. Up arrow and down arrows represent up- and down-regulation after drought treatment, respectively. **d** Coverage of parallel analysis of RNA end reads and RNA-sequencing reads on selected genes; each had two replicates. The genes *Si021866m* and *Si013398m* belong to type-I, *Si036695m* and *Si022331m* to type-II and *Si016654m* and *Si023461m* to type-IV. “+PEG” and “−PEG” represent samples with (+PEG, drought-treated) and without (−PEG, control) PEG treatment. The y-axis represents the normalized read depth

We detected 26,802 genes with uncapped transcripts in at least one of the samples according to the PARE analysis. To explore whether 5′ to 3′ mRNA degradation is associated with the drought response, we compared the degradation levels of these genes and found that 1553 genes exhibited significant changes after drought treatment, with two-thirds of them up-regulated (Fig. 1a). This result demonstrates that the process of mRNA degradation plays an important role in the drought response. For example, four late embryogenesis abundant (LEA) domain-containing protein genes were included in the list of the 10 most significantly up-regulated genes (Table 1). This finding is consistent with previous reports that LEA proteins are associated with cellular tolerance to dehydration induced by salinity, freezing or drying [41–43]. In addition, we found that genes for two MYB-family and one WRKY-family transcription factor(s), which are regulators of various plant developmental and physiological processes in response to drought stress [44, 45], were among the 10 most significantly down-regulated genes in our experiments (Table 1).

**Table 1:** Function annotations of top 10 up-regulated and down-regulated genes in PARE analysis

Transcript ID	Log_2_ (Dd/Dc)	Best rice hit name	Best rice hit define
Up-regulated			
Si037737m	9.53	Os03g19290.1	mitochondrial import inner membrane translocase subunit Tim17, putative, expressed
Si026583m	9.34	Os11g26570.1	dehydrin, putative, expressed
Si029917m	9.20	Os03g20680.1	late embryogenesis abundant protein 1, putative, expressed
Si017178m	8.96	Os02g52210.1	zinc finger, C3HC4 type domain containing protein, expressed
Si023261m	8.59	Os05g46480.1	late embryogenesis abundant protein, group 3, putative, expressed
Si016486m	8.52	Os02g15250.1	late embryogenesis abundant domain-containing protein, putative, expressed
Si021866m	8.11	Os04g57880.1	heat shock protein DnaJ, putative, expressed
Si039986m	8.09	Os10g36180.1	expressed protein
Si011162m	8.02	Os01g03320.1	BBTI2 - Bowman-Birk type bran trypsin inhibitor precursor, expressed
Si002813m	7.83	Os01g50910.1	late embryogenesis abundant protein, group 3, putative, expressed
Down-regulated			
Si022090m	−6.45	Os03g43440.1	CAMK_KIN1/SNF1/Nim1_like.17 - CAMK includes calcium/calmodulin depedent protein kinases, expressed
Si014685m	−5.83	Os08g06110.2	MYB family transcription factor, putative, expressed
Si022014m	−5.59	Os05g31730.1	transporter, monovalent cation:proton antiporter-2 family, putative, expressed
Si013398m	−5.46	Os08g06110.2	MYB family transcription factor, putative, expressed
Si030389m	−5.26	Os09g11230.1	Ser/Thr protein phosphatase family protein, putative, expressed
Si010832m	−4.75	Os11g02520.1	WRKY104, expressed
Si003491m	−4.74	Os01g21250.1	late embryogenesis abundant protein, putative, expressed
Si000941m	−4.42	Os07g35350.1	glucan endo-1,3-beta-glucosidase precursor, putative, expressed
Si005093m	−4.22		
Si014375m	−4.17	Os08g01140.1	cytochrome b561, putative, expressed

“Dc” and “Dd” represent degradation level in control sample (Dc) and drought-treated sample (Dd) revealed by parallel analysis of RNA end tags. Annotations were retrieved from phytozome. Note: Columns are blank if no corresponding data is available

### Different transcript regulation patterns during the drought response

mRNA synthesis and degradation both affect mRNA abundance. To study the connection between mRNA synthesis and degradation in transcript regulation under drought treatment, we next measured genome-wide gene expression levels using our previously published RNA-seq data [4]. A total of 2824 genes showed significant expression level changes after drought treatment, with almost equal numbers up-regulated and down-regulated. In addition, we found that 34.6 % (1126) of genes displayed significant changes in both expression and degradation levels after drought treatment (Fig. 1b), implying the existence of different transcript regulation patterns during the drought response. To reveal the overall trend of mRNA synthesis and degradation changes, we calculated the fold changes in gene expression and degradation levels for 19,814 genes identified by both RNA-seq and PARE data analysis. We uncovered a positive correlation (Pearson’s correlation: *R*^*2*^ = 0.56) between the fold-change values of transcription and degradation levels under drought conditions (Fig. 1c). We then classified these genes into nine categories with a log_2_ fold change of ±1 as the threshold, and found that 86.4 % (17,118 genes, class E in Fig. 1c) of the genes were unchanged. The remaining 2696 genes, comprising the other eight classes (classes A, B, C, D, F, G, H and I in Fig. 1c), were inferred to be involved in the drought response and regulated by either RNA transcription or RNA degradation. We further classified these drought-responsive genes into four types according to their characteristic changes (Table 2) and performed a Gene Ontology (GO) enrichment analysis using the WEGO online tool (http://wego.genomics.org.cn/cgi-bin/wego/index.pl) [46] (Additional file 4). We were thus able to aggregate genes with different transcript regulation patterns and view their functional classifications. Notably, only two genes were found to belong to type III (Table 2; Classes A and I in Fig. 1c), in which transcript and uncapped transcript abundance showed opposite trends. As a consequence, no further analysis was performed on type-III genes.

**Table 2:** Four different transcript regulation patterns in drought stress response

Type	Gene number	Change after PEG-treatment
I	671	D↑R↑
	453	D↓R↓
II	260	D↑R-
	63	D↓R-
III	2	D↑R↓
	0	D↓R↑
IV	466	D-R↑
	781	D-R↓

D: uncapped mRNA abundance indicated by parallel analysis of RNA end tags; R: transcript abundance indicated by RNA-Seq reads. Genes with log_2_ (fold change) ≥ 1 and a *P*-value < 0.001 were considered as up regulated genes and genes with log_2_ (fold change) ≤ − 1 and a *P*-value < 0.001 were considered as down regulated genes. Genes with −1 < log_2_ (fold change) < 1 and a *P*-value > 0.001 were designated as unchanged

Type-I genes (Table 2; classes C and G in Fig. 1c), which were characterized by transcript and uncapped transcript abundances changing in the same direction after drought treatment, were enriched in catalytic and various oxidation-related enzymes (oxidoreductase, antioxidants and peroxidase) in the molecular function category as well as the biological process subcategories ‘metabolic process’, ‘response to stimulus’ and ‘response to stress’ (Additional file 4). PARE and RNA-seq read coverage is shown for two examples in Fig. 1d. One of these genes, *Si021866m*, encodes a DNAJ heat shock N-terminal domain-containing protein. Its homologous gene in *Arabidopsis thaliana* plays an important role in cellular stress sensors [47]. The other example, *Si013398m,* encodes a transcription factor belonging to the MYB family reported to play crucial roles in plant responses to abiotic stress [44, 48].

Genes in the type-II category (Table 2; classes B and H in Fig. 1c), comprising genes showing significant changes in uncapped transcript abundance after drought treatment but no changes in transcript abundance, were typified by *Si036695m,* a No Apical Meristem (NAC) transcription factor, and *Si022331m*, a bZIP transcription factor (Fig. 1d). Genes related to transcription factors, transcription regulators, pigmentation and regulation processes (under the biological and cellular metabolic categories) were overrepresented in this group (Additional file 4).

Type-IV genes (Table 2; classes D and F in Fig. 1c) showed significant changes in transcript abundance while their uncapped transcripts remained unchanged after drought treatment. Genes associated with membranes were enriched in the cellular component category, and various important catalytic activities (hydrolase, lyase and isomerase) in the molecular function category and ‘response to chemical stimulus’ and ‘oxidation reduction’ in the biological process category were heavily represented. *Si016654m*, a representative of type IV (Fig. 1d), encodes an arginine decarboxylase (ADC) protein. Previous studies have found that ADC is involved in responses to salt, drought and other abiotic stresses [49–51], with other investigations revealing that transgenic ADC rice plants show increased biomass under salinity-stress conditions compared with non-transformed controls [52]. *Si023461m*, another type-IV example, encodes ribulose bisphosphate carboxylase, the crucial enzyme in photosynthesis and photorespiration. Sharkey et al. [53] showed that mild water stress affects ribulose bisphosphate carboxylase activity in intact leaves.

Detailed functional annotations for type-I, −II and -IV genes are given in Additional file 5. Taken together, our data demonstrate that different transcript regulation patterns exist during the drought response and may be correlated with gene function.

### Sequence characteristics correlated with different mRNA decay patterns

**Fig. 2:**
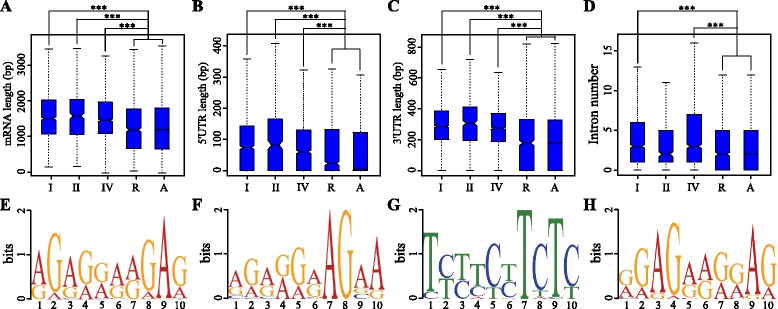
Gene transcript features and sequence motifs contributing to different mRNA decay patterns. **a**-**d** A display of mRNA length, 5′ UTR length, 3' UTR length and number of introns for different gene types. “I”, “II”, “IV”, “R” and “A” represent type-I, −II, −IV, randomly selected genes and all genes, respectively. “***” indicates statistically significant difference at *P-*value < 0.001 (Student’s wilcox-test). **e-h** Enriched motifs (*E*-value < 0.001) in the 5' UTRs of type-I (**e**), type-II (**f** and **g**) and type-IV (**h**) genes

Sequence characteristics have been reported to contribute to uncapped mRNA abundance [15, 16]. To reveal the characteristics of mRNAs with different decay patterns, we calculated the mRNA lengths, GC contents, minimal folding free energy indexes (MFEIs) [54] of secondary structures, untranslated region (UTR) lengths and intron numbers for type-I, −II and -IV genes (Fig. 2 and Additional file 6). The results showed that the mean lengths of 5′ UTRs, 3′ UTRs and mRNAs of type-I, −II and -IV genes were significantly greater (*P* < 0.001) than those of all genes and 898 (average number of I, II and IV genes) randomly selected genes (Fig. 2a–c). Moreover, the mean MFEI values of 5′ UTRs, 3′ UTRs and mRNAs of type-I, −II and -IV genes were significantly lower than those of all genes and randomly selected genes (Additional file 6). In contrast, no significant differences were found for the mean GC contents of 5′ UTRs and 3′ UTRs among these gene groups (Additional file 6). Overall, the lengths and MFEIs of UTRs and mRNAs correlated with the structural features of genes involved in the drought response.

Recent studies have revealed a relationship between introns and mRNA stability [55, 56]. In our study, the average number of introns in type I and IV genes was higher than in all genes and randomly selected genes (*P* < 0.001), whereas no significant differences were found between type-II genes and the latter gene categories (Fig. 2d). This result indicates that the number of introns has an obvious influence on drought-responsive genes, and this effect is related to mRNA synthesis regulation rather than degradation regulation.

Previous research has shown that enriched motifs in 5′ and 3′ UTR regions can affect mRNA stability [15, 16, 57]. We therefore used an integrated motif-discovery program (MEME) [58, 59] to identify possible motifs located in the 5′ and 3′ UTRs of type-I, −II and -IV gene transcripts. We identified significantly enriched motifs (*E*-value < 0.001) in the mRNA 5′ UTRs: one in type I (Fig. 2e), one in type IV (Fig. 2h) and two in type II (Fig. 2f and g), implying the existence of some specific regulators or regulatory mechanisms in their 5′ UTRs. Next, we analyzed these enriched motifs using Tomtom tool. The best-matched motif to ‘[AG]G[AG][GA][GA][AG][AG]GA[GA]’ (Fig. 2e) was RNCMPT00044 (*P* = 2.31528e-05). The protein Poly (rC)-binding protein 2 (PCBP2) was reported to bind the RNCMPT00044 motif [60] and appears to be multifunctional [61, 62]. RNCMPT00019 was the best-matched motif to ‘[AG][GA][AG][GA][GA][AG]AGAA’ (Fig. 2f) (*P* = 0.002) and RNCMPT00088 was the best-matched motif to both ‘T[CT][TC][TC][CT][TC]TCT[CT]’ (Fig. 2g) (*P* = 2.16363e-06) and ‘[GA][GA]AG[AG][AG][GA][GA]A[GA]’ (Fig. 2h) (*P* = 6.99643e-05). The binding protein for both RNCMPT00019 and RNCMPT00088 is serine/arginine-rich splicing factor 10 (SRSF10) [60]. SRSF10 is known to function as a sequence-specific splicing activator [63] and can promote both exon inclusion and exclusion in chicken cells [64]. This suggests that some RNA-binding proteins may play a role in the behavior of these gene classes. In contrast, no significantly enriched motifs were found in the 3′ UTRs of type-I, −II and -IV genes.

### Identification of endogenous miRNA cleavage targets

**Fig. 3:**
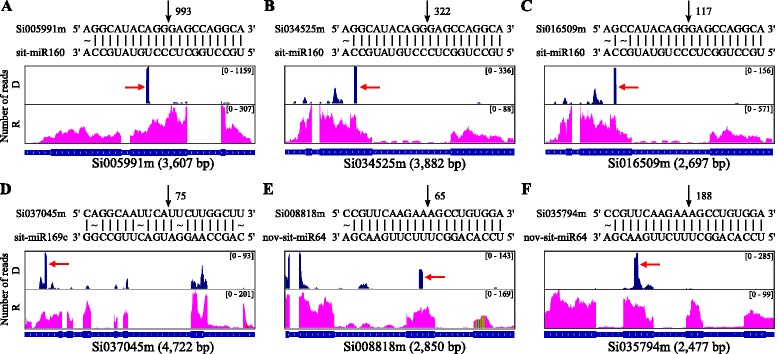
**a-f** Examples of confidently identified miRNA-directed cleavage. The complementary patterns of miRNA sequences and partial sequences of the target mRNAs are shown in the upper part of the figure and the numbers from parallel analysis of RNA end tags corresponding to cleavage sites are indicated by vertical arrowheads. “D” and “R” represent coverage of parallel analysis of RNA end tags (D) and RNA-sequencing reads (R) on selected miRNA targets. The mapped tags in “D” with the frequency at the position between bases 10 and 11 (from the miRNA 5') of the inset miRNA target alignment are indicated by red vertical arrowheads. Full details of all confidently identified miRNA targets are shown in Additional file 6. The sequences used for this figure came from the control sample. The number of reads mapping to each gene is indicated at the upper right

In plants, miRNAs play key roles in many developmental events and regulate their target transcripts through two modes of action: degradation and translation inhibition [65–67]. In this study, PARE-seq was used to identify the cleavage sites of targets mediated by miRNA-programmed RISCs [24]. Using the PARE data, we identified 385 putative miRNA-guided cleavages in foxtail millet (Additional file 7); six prominent examples were selected for detailed discussion (Fig. 3). miR160 guides cleavage within the coding regions of *Si005991m* (993 reads across the Dc PARE libraries, Fig. 3a), *Si034525m* (322 reads, Fig. 3b) and *Si016509m* (117 reads, Fig. 3c). The proteins encoded by *Si005991m*, *Si034525m* and *Si016509m* are homologs of *A. thaliana* auxin response factor 16 (ARF16). A previous study determined that ARF16, targeted by miR160, controls root cap cell formation in *A. thaliana* [68]. miR169c guides cleavage within the coding region of *Si037045m* (75 reads across the Dc PARE libraries, Fig. 3d), while nov-sit-miR64 guides cleavage of *Si008818m* (65 reads, Fig. 3e) and *Si035794m* (188 reads, Fig. 3f). We found that the Si008818m and Si035794m proteins contain the same characteristic regions, namely QLQ (Gln, Leu and Gln) and WRC (Trp, Arg and Cys) domains, as *A. thaliana* growth-regulating factor proteins (AtGRFs). AtGRFs are involved in cell expansion in leaf and cotyledon tissues [69]. Examination of PARE-seq and RNA-seq reads mapping to the six miRNA target transcripts (Fig. 3) revealed a prominent cluster of reads at predicted cleavage locations in the PARE libraries, while no such pattern emerged in the RNA-seq library. These results, which reveal that miRNA-mediated degradation is the main pathway of mRNA degradation for some miRNA targets, were visualized using the Integrative Genomics Viewer [70].

### Insights gained into miRNA precursor metabolism

In plants, most primary miRNAs (pri-miRNAs) that are transcribed from miRNA genes by RNA polymerase II undergo two sequential cleavages by DCL1 to yield an RNA duplex containing the mature miRNA and miRNA* sequences [71, 72]. The locations of PARE tags mapped to the precursor can provide valuable hints to help reveal the details of the DCL1-guided two-step cleavage action on miRNA precursors [33].

**Fig. 4:**
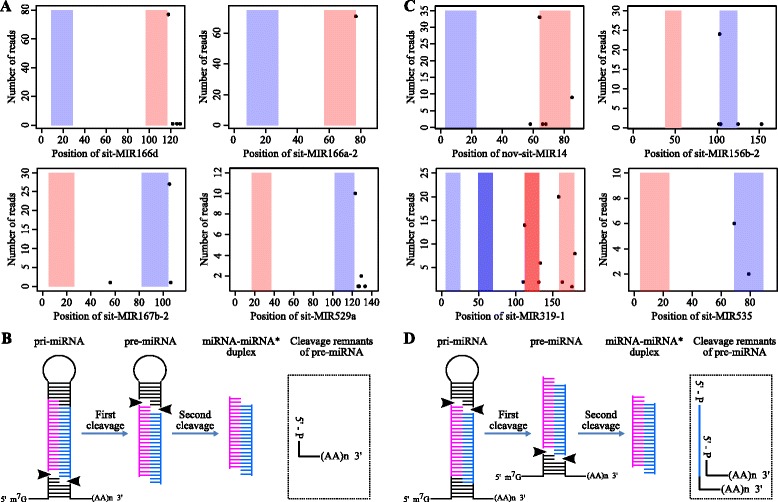
PARE tags mapping to foxtail millet miRNA hairpins. **a** and **c** Examples of “loop-last” (**a**) and “loop-first” (**c**) miRNA precursor processing. **b** and **d** A diagram of “loop-last” (**b**) and “loop-first” (**d**) processing. Regions within the pink and blue bars in (**a**) and (**c**) indicate the positions of the miRNA and miRNA* in the precursor, respectively. Two distinct miRNA/miRNA* duplexes were generated from sit-MIR319-1 and the two darker bars in sit-MIR319-1 indicate the miRNA/miRNA* duplexes of nov-sit-miR149. The read count at each position is indicated as a scatter plot

To probe the patterns of miRNA hairpin processing in foxtail millet, the PARE reads in our datasets were mapped to 301 annotated [73] foxtail millet miRNA precursors (pre-miRNAs). Of the annotated pre-miRNAs, 114 (37.8 %) had one or more matching PARE tags (Additional file 8). The matching PARE tags were mainly mapped to precursors in one of two places: either the lower or upper cleavage site of the stem-loop 3′ arm. As shown in Fig. 4a, the matching PARE tags were mainly mapped to sit-miR166d, sit-miR166a-2, sit-miR167b-2 and sit-miR529a precursors at the lower cleavage site of the stem-loop 3′ arm; this indicates that these pre-miRNA hairpins could be processed by DCL1 via the classical loop-last mechanism [33] in which the first cleavage of pre-miRNA hairpins occurs at the loop-distal position (Fig. 4b). As shown in Fig. 4c, in contrast, matching PARE tags were mainly mapped to nov-sit-miR14, sit-miR156b-2, sit-miR319-1 and sit-miR535 precursors at the upper cleavage site of the stem-loop 3′ arm. This matching position implies that these pre-miRNAs are processed by DCL1 via an unusual loop-first mode [34, 74] in which the first cleavage of pre-miRNA hairpins occurs by precise processing at loop-proximal sites (Fig. 4d). It is noteworthy that this loop-first sequential processing of pre-miR319 family hairpins (sit-miR319-1 in Fig. 4a and sit-miR319-2 in Additional file 9) is also seen in *A. thaliana*, *Physcomitrella* and rice, and can generate two distinct miRNA/miRNA* duplexes [34, 74]. We also discovered that all miR156 family precursors identified in our study (sit-miR156b-2 in Fig. 4a and sit-miR156a-1, sit-miR156b-3 and sit-miR156d-2 in Additional file 9) are processed via the loop-first mechanism, whereas all identified miR166 family precursors (sit-miR166d and sit-miR166a-2 in Fig. 4b and sit-miR166a-1, sit-miR166a-3 and sit-miR166a-5 in Additional file 9) are associated with the loop-last mechanism.

In pre-miRNA hairpin processing, DCL1-mediated cleavage occurs on each strand of the stem region [75]. The resulting 3′ cleavage products with poly (A) tails can be cloned by PARE high-throughput sequencing. If first-step cleavage occurs on both arms simultaneously, only cleavage signals mapped to the 3′ arm of pre-miRNA hairpins will be detected. If first-step cleavage occurs on both arms non-simultaneously, however, cleavage signals mapped to both the 3′ and 5′ arms of pre-miRNA hairpins will be identifiable in the PARE data. Our PARE data showed hardly any cleavage signals mapped to the stem-loop miRNA 5′ arm (Fig. 4 and Additional file 9), indicating that first-step cleavage occurs on both arms, most likely simultaneously, during the processing of most foxtail millet miRNA hairpins.

### Distinct mRNA decay patterns among gene functional classes

**Fig. 5:**
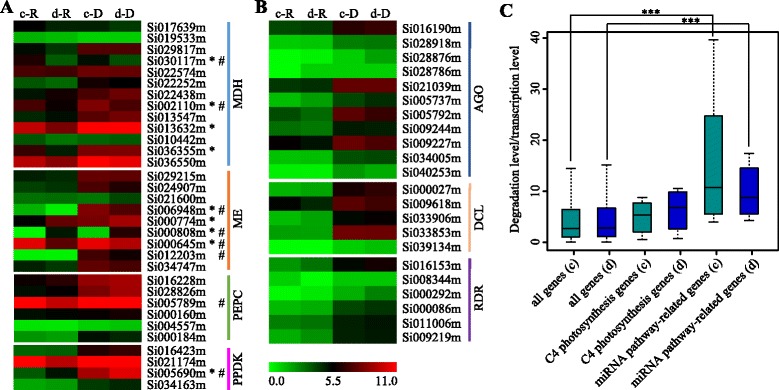
Expression pattern of C_4_ photosynthetic-related genes and miRNA pathway-related regulators under drought stress. **a** and **b** Heatmaps showing the degradation level (RPM) and transcription level (RPKM) of genes encoding C_4_ photosynthesis pathway-related enzymes (**a**) and miRNA pathway-related regulators (**b**). Asterisks represent significant changes in transcription level after drought stress. Number signs represent significant changes in degradation level after drought stress. “c-R” and “d-R” represent transcription levels in the control (c-R) and drought-treated (d-R) samples revealed by RNA-seq. “c-D” and “d-D” represent degradation levels in the control (c-D) and drought-treated (d-D) samples revealed by PARE-seq. **c** The distribution of the ratio of relative uncapped mRNA abundance (RPM) versus total mRNA abundance (FPKM). c: control sample; d: drought-treated sample. “***” indicates statistically significant difference at *P*-value < 0.001 (Student’s Wilcox-test)

As a diploid Panicoid crop species, foxtail millet uses C_4_ pathway photosynthesis. Although the leaves of C_4_ crops have increased nitrogen and water use efficiencies compared with C_3_ species [76], virtually nothing is known about how the pathway is regulated under drought stress. In C_4_ photosynthesis, carbon is shuttled as a C_4_ acid from the mesophyll to the bundle sheath cells to create a CO_2_ pump through a series of enzyme catalytic reactions. These enzymes include phosphoenolpyruvate carboxylase (PEPC), malate dehydrogenase (MDH), NADP-malic enzyme (ME) and pyruvate phosphate dikinase (PPDK). Using Phytozome, we found 32 C_4_ carbon shuttle enzyme (PEPC, MDH, PPDK and ME) genes in foxtail millet. To better understand the effect of drought on C_4_ enzymes, we analyzed the transcription and degradation of these 32 C_4_-related genes after PEG-induced drought stress (Fig. 5a). The transcript abundance of nine genes (four MDH genes, four ME genes and one PPDK gene) and the degradation abundance of eight genes (two MDH genes, four ME genes, one PPEC gene and one PPDK gene) showed significant changes after drought treatment. Among these drought-responsive genes, six displayed significant changes at both the transcription and degradation levels after drought treatment. These results suggest that various transcriptional and degradation regulatory mechanisms operate in C_4_-related genes under drought stress and may function to regulate water use efficiency in foxtail millet.

We also analyzed the effect of drought on relevant core regulators in the miRNA pathway, such as AGO, DCL and RNA-dependent RNA polymerase gene family members. Unlike C_4_-related genes, none of the 22 miRNA pathway-related regulator genes showed significant changes after drought treatment at the transcription or degradation levels (Fig. 5b). Interestingly, we found that the largest numbers of transcripts of these miRNA pathway-related regulators were enriched in uncapped forms (Fig. 5c). The distribution of relative uncapped to total mRNA ratios was found to be significantly biased (*P* < 0.001), which is consistent with results reported previously in Arabidopsis [15].

## Discussion

### Different transcript degradation patterns were revealed during drought stress responses

To examine both gene synthesis and gene degradation, which were revealed respectively by RNA-seq and PARE-seq, genes were divided into four groups according to their change patterns after drought stress (Table 2).

Type-II genes (Table 2; classes B and H in Fig. 1c) could not be detected by analysis of RNA-seq data alone, as the amounts of synthesized mRNA were unchanged by the environmental stress conditions. GO analysis (Additional file 4) and detailed functional annotation (Additional file 5) revealed that many of the type-II genes belonged to diverse families of transcription factors such as WRKYs, MYBs, bZIPs and NACs. Many of these transcription factors play important roles in responses to drought stress [44, 45, 48, 77, 78]. Two significantly enriched motifs were identified in the 5′ UTRs of type-II gene mRNAs (Fig. 2f and g), implying the existence of some specific regulatory mechanism in this gene group. In contrast, the amounts of degraded type-IV gene (Table 2; classes D and F in Fig. 1c) mRNAs remained unchanged after drought treatment, whereas synthesized mRNA quantities showed obvious alterations. In the GO analysis, catalysis-related genes, such as hydrolases, isomerases, lyases, oxidoreductases and peroxidases (Additional file 4), were obviously over-represented among type-IV genes. Peroxidases are widely accepted as ‘stress enzymes’ [79]. Oxidoreductase can decompose H_2_O_2_ to water and molecular oxygen and is one of the key enzymes involved in the removal of toxic peroxides [80, 81]. Induction of the activity of these enzymes has been documented under a variety of stressful conditions, such as water stress [82–84], chilling [85] and salinity [86], implying that these type-IV genes may serve as an intrinsic defense tool to resist drought stress in foxtail millet.

In type-I genes (Table 2; classes C and G in Fig. 1c), synthesized and degraded mRNA amounts followed similar trends after drought treatment. The GO enrichment analysis (Additional file 4) indicated that these genes were enriched in catalytic and various oxidation-related enzymes, suggesting that these types of genes may be important regulators of reactive oxygen species removal to maintain redox balance under drought stress conditions. The amounts of synthesized and degraded mRNAs showed opposite trends in type-III genes (Table 2; Classes A and I in Fig. 1c) following drought treatment. Unexpectedly, we detected only two type-III genes, for which changes in intact mRNA levels were enhanced by opposing changes in synthesis and degradation. However, in research on *Brachypodium distachyon* under cold stress, there were 1166 genes in type III (in which changes in transcript and uncapped transcript abundance showed opposite trends after cold treatment), but no obvious functional enrichment among these genes was found in GO analysis [16]. There are three possible reasons for this difference: (i) plants may have different regulation patterns in response to different abiotic stresses (cold and drought), (ii) different plants, e.g., *B. distachyon* (C_3_) and foxtail millet (C_4_), have different adaptation mechanisms in response to environmental stress, and (iii) plants have different regulatory mechanisms in response to different periods of abiotic stress (cold treatment for 24 h and drought treatment for 7 h). Perhaps a strong organismal response was not needed because of the short (7-h) duration of the drought treatment in our study.

### miRNA targets

Using the PARE data in this study, we identified 385 putative miRNA-guided cleavages in foxtail millet (Additional file 7). Thus far, the transcripts of eight protein-coding targets of miRNA-mediated cleavage have been confirmed by gene-specific 5′ RACE in foxtail millet [73, 87]. Among these eight miRNA targets, seven (*Si005991m*, *Si016509m*, *Si034525m*, *Si016508m*, *Si001804m*, *Si006975m* and *Si025305m*) were found in our PARE data. A previous 5′ RACE analysis revealed that the *Si016508m* gene has two cleavage sites (at bp-positions 1085 and 1082) mediated by different miRNAs [73]. We also identified these two breakpoints in the *Si016508m* gene in our PARE analysis (Additional file 7). These results indicate that the miRNA-guided cleavages identified by the PARE analysis are genuine. A functional miRNA is expected to regulate target transcripts through two modes of action, either degradation or translation inhibition [13, 66, 67]. Because of the absence of detectable slicing, the PARE analysis was unable to find targets of miRNAs that act by repressing translation [30].

In a previous study, 43 known miRNAs and 212 novel miRNAs were identified in foxtail millet [73]. In our PARE analysis, we confirmed the targets of 80 % (34) of these known miRNAs, but only 34 % (73) of the novel ones (Additional file 7). Compared with known sit-miRNAs, nov-sit-miRNAs have been reported to have relatively lower expression levels and to exhibit higher tissue-specific expression [73]. Thus, we may have identified smaller numbers of targets of known and especially novel miRNAs because we did not analyze many developmental stages or different tissues.

Analysis of miRNA target expression after PEG-induced drought stress revealed that 50 miRNA targets were associated with different decay patterns during drought response (Additional file 10). The expression levels of some miRNA targets have been reported to be significantly altered after drought stress [50, 77, 88, 89]. For example, NAC genes targeted by miR164 negatively regulate drought resistance in rice [77], and nuclear factor Y, the target of miR169, is regulated transcriptionally and post-transcriptionally by miR169 to promote drought resistance in Arabidopsis [89]. In our study, the NAC protein genes *Si017567m* and *Si010553m* are also targets of miR164 and involved in response to drought stress. And *Si037045m*, a nuclear factor Y gene, is also targeted by miR169 and strongly induced by drought stress (Additional file 10). In addition, previous research revealed that the expression levels of sit-miR156, sit-miR160 and sit-miR397 were significantly altered after PEG-induced drought stress in foxtail millet [90]. Here, we found some of their targets, such as *Si001804m* (targeted by sit-miR156), *Si016509m* (targeted by sit-miR160) and *Si001277m* (targeted by sit-miR397), were associated with different decay patterns during the drought stress response (Additional file 10).

Besides their possible involvement in plant drought stress resistance, the miRNA targets identified in this study also play fundamental roles in plant growth and development. For example, GRFs targeted by nov-sit-miR64 have been shown to play an important role in cell expansion in leaf and cotyledon tissues in *A. thaliana* [69]*.* SPLs and AP2, targeted by miR156 and miR172, respectively, are responsible for the juvenile to adult transition in Arabidopsis [91].

### miRNA precursor metabolism

The critical step of miRNA biogenesis is the precise processing of miRNA/miRNA* duplexes from precursor miRNA hairpins. Our PARE data suggest that both processing mechanisms exist in foxtail millet; some miRNA biogenesis was consistent with loop-last processing (Fig. 4a), whereas the precise processing of some miRNA precursors followed the unusual loop-first mode (Fig. 4b). We also observed that several different small RNAs originated from the same miRNA precursors. One such example is nov-sit-miR110 (conserved with miR159 in Arabidopsis) and nov-sit-miR155 (Additional file 9). This observation is consistent with prior research [92] demonstrating that the miR159 precursor can generate more than one mature miRNA in Arabidopsis. A similar example was found in the pre-miR319 family (Fig. 4 and Additional file 9), where two distinct miRNA/miRNA* duplexes were released from pre-miR319, both through loop-first processing (Fig. 4 and Additional file 9). Previous research has also demonstrated the occurrence of precise loop-first processing of an artificially generated miRNA with an Arabidopsis miR319 backbone [86]. One possible explanation for these observations is that one or more *cis*-acting hairpin features may direct DCL1 first-cleavage of miRNA hairpins at the loop-proximal position that is spatially separated from the loop-distal regions [34]. Identifying the features that direct both loop-last and loop-first modes of precise plant miRNA hairpin processing is an important goal for future research.

In addition to revealing sites corresponding to the remnants of the DCL-catalyzed cut, a previous study uncovered the existence of other cleavage signals in the middle of either miRNA- or miRNA*-coding loci on pre-miRNAs that are mediated by miRNA or miRNA* self-regulation [25]. In our study, we detected cleavage signals in the middle of miRNA- or miRNA*-coding loci in sit-miR535 (Fig. 4b), nov-sit-miR144-2 and nov-sit-miR120 (Additional file 9) precursors in foxtail millet. This finding is consistent with related observations in rice [28] and Arabidopsis [33] that imply that miRNA- or miRNA*-mediated self-regulation of certain miRNA precursors, although not widespread, exists in various organisms.

Some unexpected cleavage signals were also detected in several miRNA hairpins (Additional file 9C). For example, many different cleavage signals were detected in the region where mature nov-sit-miR121-1-5p and nov-sit-miR121-1-3p were located, while a few cleavage signals were found in other locations in the nov-sit-miR121-1 precursor. In addition, many different cleavage signals were detected in all nov-sit-miR35, nov-sit-miR82 and nov-sit-miR111 precursors. Similar unexpected results have also appeared in previous studies [28, 33] and could not be caused by methodological problems [28]. Although the currently understood model of miRNA biogenesis has been well studied [12, 28, 33, 34, 92], some novel processes not yet revealed must be responsible for these unexpected results and need further study.

## Conclusions

In this study, mRNA synthesis and degradation, revealed respectively by RNA-seq and PARE data, were both taken into consideration in a global exploration of gene expression regulation under drought stress. This global analysis revealed new insights into gene expression under drought stress, confirmed many known regulatory mechanisms, and provided a window into many additional potentially novel pathways. Specific degradation patterns, such as miRNA mediation of target degradation and DCL1-mediated miRNA biogenesis were uncovered. Our results will not only deepen the understanding of mRNA degradation under stress conditions, but will also allow further insights into many targets of both known and novel miRNAs. Finally, these findings shed light on miRNA-precursor processing mechanisms in gramineous crops and biofuel grasses, which have close evolutionary relationships with foxtail millet.

## Methods

### Plant material and sequencing

Foxtail millet inbred line Yugu1 [35] was used in this study. Seeds of Yugu1 were germinated on a moist filter containing two layers of damp filter paper and incubated at 28 °C for 24 h. The germinated seedlings were then grown for 9 days in pots filled with a 1:1 mixture of nutrient soil: vermiculite under an illuminating incubator (28 °C day/20 °C night, 14-h photoperiod and 70 % relative humidity). The seedling roots were gently washed, transferred to 1/4 Hoagland’s solution, and allowed to grow for another 5 days. The aerial parts of seedlings (shoots), either subjected to drought stress (20 % PEG 6000 for 7 h) or untreated, were pooled and used for construction of the Dc and Dd libraries, respectively. The drought treatment was performed according to [4]. Total RNA was extracted from the 14-day-old Yugu1 seedlings using Trizol reagent (Invitrogen, USA) following the manufacturer’s protocol. PARE libraries were prepared according to [24], but with modifications described in [93]. Detailed descriptions of each step are provided in Additional file 1. The sequencing libraries were analyzed on a HiSeq 2000 sequencer (Illumina, USA) to produce 50-bp single reads. The generated raw reads have been deposited in NCBI’s SRA database under the accession number SRP061964.

### PARE analysis and RNA-seq analysis

The foxtail millet genome sequence was downloaded from the Phytozome database (Sitalica_164_hardmasked.fa) [35, 94]. To analyze the PARE sequencing data, sequences corresponding to known tRNAs, rRNAs, small nucleolar RNAs, small nuclear RNAs and repeats/transposons were first removed [39, 40], and the remaining sequences were mapped to the foxtail millet genome using bowtie [95], with one mismatch allowed. Low-quality reads were excluded from subsequent analyses. PARE reads mapping to multiple positions were divided among the different positions [16]. An in-house developed Perl script was used to identify the total reads for specific transcripts. To compare specific transcripts, the mapped reads were enumerated and normalized against the total count of genome-mapped reads, reported as RPM, for each respective library. To assess the biological replicates, the log_2_ normalized data (RPM value + 1) were used to calculate correlation coefficients. Because the correlation between biological replicates was high (average *R*^*2*^ = 0.97), we took the average RPM as the expression quantity. Genes with an adjusted *P*-value < 0.001, as determined by the DEGseq [96] software, and log_2_(fold change) ≥ 1 were considered up-regulated, while those with log_2_(fold change) ≤ −1 were considered down-regulated. Genes with −1 < log_2_(fold change) < 1 and a *P*-value > 0.001 were designated as unchanged.

To analyze the RNA-seq data, our previous analysis method was used [4]. After low quality reads were removed, Illumina sequencing reads were mapped to the foxtail millet genome using TopHat v2.0.4 [97], allowing two mismatches for the 100 bp reads. Then, the SAM files generated by TopHat were used as inputs for the Cufflinks software [97] and their expression levels (FPKM, fragments per kilobase of transcript per million fragments) were calculated with the foxtail millet genome annotation file. Genes with log_2_ (fold change) ≥ 1 and a *P*-value < 0.001 were considered up-regulated and genes with log_2_ (fold change) ≤ −1 and a *P*-value < 0.001 were considered down-regulated. Genes with −1 < log_2_ (fold change) < 1 and a *P*-value > 0.001 were designated as unchanged.

### Gene ontology annotation

GO analysis was performed using WEGO [46]. The annotation frequency for each gene type was compared with corresponding annotations for the entire foxtail millet genome [35]. The Pearson chi-square test was used for statistical analysis. GO categories that showed significant (α = 0.05) enrichment were analyzed and displayed in the WEGO output histogram.

### Analysis of sequence features

To analyze the features of mRNAs with different decay patterns, foxtail millet transcript sequences (release 164) and annotation data were downloaded from Phytozome [98]. The MFE value of the MFEI (MFE/(length of RNA sequence) × 100/(G + C)%) for secondary structures was calculated using RNAfold [99]. All other pattern searches and calculations were performed using custom scripts in Perl and R. Enriched motifs were identified using the MEME software [59] with *E*-value < 0.001. The Tomtom tool in MEME Suite (http://meme-suite.org/tools/tomtom) was used to determine whether there were RNA-binding proteins in the analyzed gene classes.

### miRNA target identification and miRNA precursor metabolism analysis

The classification of miRNA target categories was performed using the PAREsnip [100] pipeline based on PARE sequences (Dc library), foxtail millet transcripts (release 164) and miRNA sequences as inputs. PARE sequences (Dc library) matching miRNA hairpins according to BLASTN were assessed by comparisons of their respective genomic coordinates (Additional file 8). Foxtail millet miRNA and miRNA hairpin sequences were obtained from a previous publication [73]. Detailed information for the miRNAs discussed in this work is provided in Additional file 11.

## Additional files

Additional file 1.
**Overview of the PARE-seq method for the isolation of uncapped mRNA.** (PDF 80 kb) Additional file 2.
**Summary of PARE analysis.** (XLSX 9 kb) Additional file 3.
**Correlation between PARE biological replicates.** We calculated the expression level (RPM) for each replicate separately and compared them to one another. The normalized data of log_2_ (RPM value + 1) was used to calculate the correlation coefficient and the correlation between the biological replicates was high (average *R* ^*2*^ = 0.97). Dd: the PARE libraries of drought treatment group, Dc: the PARE libraries of control group. (PDF 65 kb) Additional file 4.
**GO functional enrichment analysis for different decay pattern mRNA.** Gene Ontology (GO) analysis was performed for the type I, II, IV genes using WEGO which organizes information for cellular component categories, molecular function and biological process. The Pearson Chi-square test was used for statistical analysis. GO categories that show a significant (α =0.05) enrichment were analyzed and displayed here. (PDF 396 kb) Additional file 5.
**Fold change and function annotations of four types of genes with different decay.** Annotations were retrieved from phytozome. Note: Columns are blank if no corresponding data is available. (XLSX 252 kb) Additional file 6.
**Gene transcript features to different mRNA decay patterns.** Analyzed GC content and MFEI value of mRNA, 5' UTR and 3' UTR for type-I, −II, −IV, randomly selected genes and all genes in foxtail millet. “R” represented randomly selected genes, “A” represented all genes, “***” indicate statistically significant differences at *P* value < 0.001 (Student’s wilcox-test). (PDF 257 kb) Additional file 7.
**miRNA/target interactions identified in the degradome library by PAREsnip and function annotations of targets.** Annotations were retrieved from phytozome. Note: Columns are blank if no corresponding data is available. (XLSX 40 kb) Additional file 8.
**PARE signatures mapping to foxtail millet miRNA hairpins.** (XLSX 34 kb) Additional file 9.
**Foxtail millet PARE tags matching miRNA hairpins.** (A) “Possible loop-last” processing of miRNA hairpins. (B) “Loop-first” processing of miRNA hairpins. (C) Unexpected cleavage signals in several miRNA hairpins. Regions within the pink and blue bars indicate the positions of the miRNA and miRNA* in the precursor, respectively. The read count at each position is indicated as a scatter plot. (PDF 356 kb) Additional file 10.
**Fold change and function annotations of miRNA targets which are response to drought.** Annotations were retrieved from phytozome. Note: Columns are blank if no corresponding data is available. (XLSX 14 kb) Additional file 11.
**The detail information about miRNAs which have discussed in this work.** (XLSX 14 kb)

## Abbreviations

miRNA: microRNA
RNA-seq: RNA-sequencing
PARE: Parallel analysis of RNA ends
RISC: RNA-induced silencing complex
RACE: Rapid amplification of cDNA ends
DCL: Dicer-like enzyme
Dd: The PARE libraries of drought treatment group
Dc: The PARE libraries of control group
RPM: Reads per million
LEA: Late embryogenesis abundant
GO: Gene ontology
NAC: No Apical Meristem
ADC: Arginine decarboxylase
MFEI: Minimal folding free energies index
UTR: Untranslated region
RISCs: RNA-induced silencing complexes
PCBP2: Poly (rC)-binding protein 2
SRSF10: Serine/arginine-rich splicing factor 10
ARF16: Auxin response factor 16
QLQ: (Gln, Leu, Gln)
WRC: (Trp, Arg, Cys)
AtGRF: *A. thaliana* growth-regulating factor
pri-miRNA: Primary miRNA
pre-miRNA: miRNA precursor
PEPC: Phosphoenolpyruvate carboxylase
MDH: Malate dehydrogenase
ME: NADP-malic enzyme
PPDK: Pyruvate phosphate dikinase
AGO: Argonaute
RDR: Dependent RNA polymerase
NF-Y: Nuclear factor Y
