# Supplementary figures and images for: Global analysis of uncapped mRNA changes under drought stress and microRNA-dependent endonucleolytic cleavages in foxtail millet
Source: BMC Plant Biol. 2015 Oct 6;15:241. doi: 10.1186/s12870-015-0632-0 (PMC4594888; doi:10.1186/s12870-015-0632-0)

# Additional file 1: Overview of the PARE-seq method for the isolation of uncapped mRNA

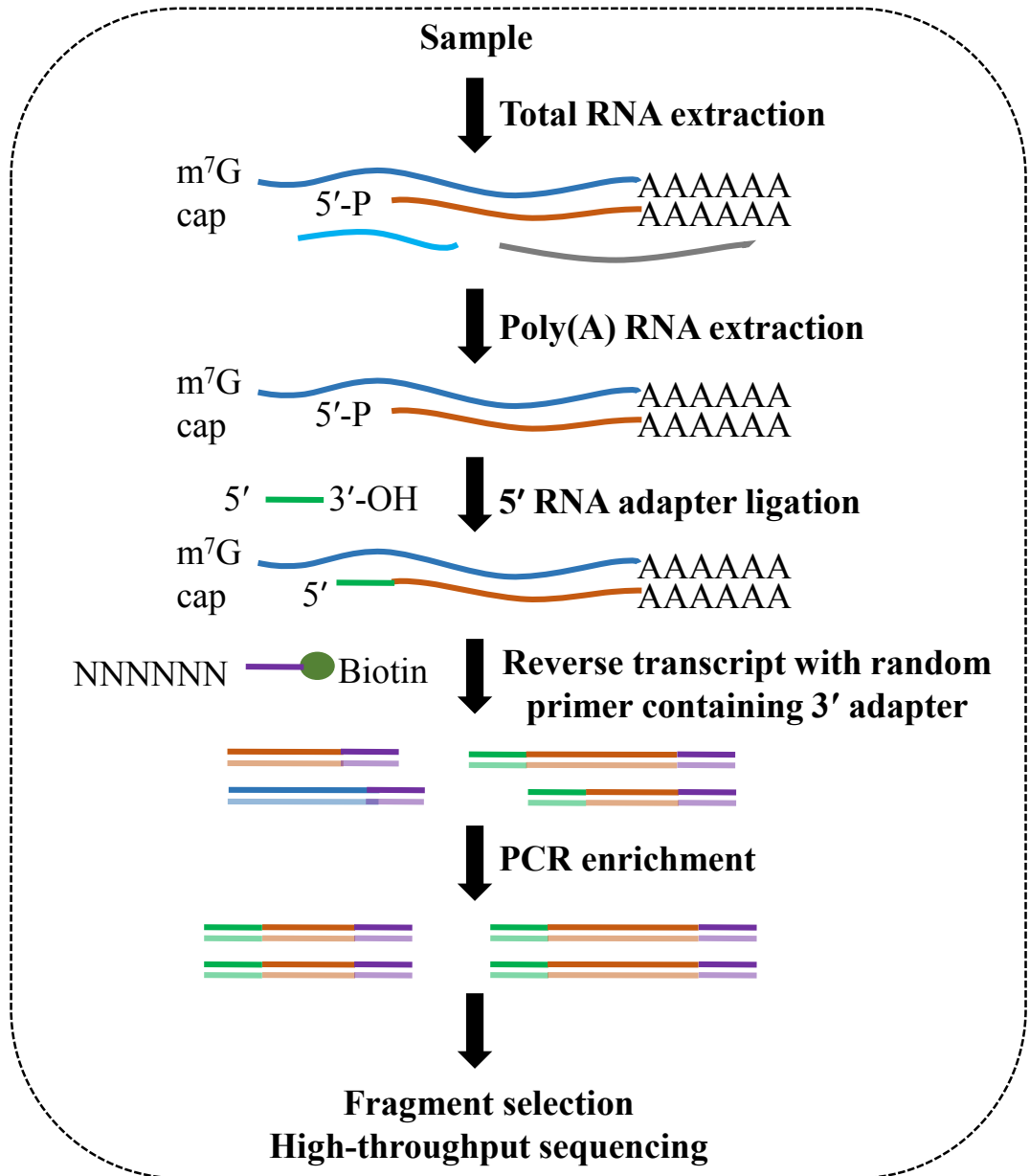

Supplement: Additional file 1. — Overview of the PARE-seq method for the isolation of uncapped mRNA. (PDF 80 kb) [file 12870_2015_632_MOESM1_ESM.pdf]
