# Supplementary material for: Global analysis of uncapped mRNA changes under drought stress and microRNA-dependent endonucleolytic cleavages in foxtail millet
Source: BMC Plant Biol. 2015 Oct 6;15:241. doi: 10.1186/s12870-015-0632-0 (PMC4594888; doi:10.1186/s12870-015-0632-0)

Additional file 3: Correlation between PARE biological replicates.

We calculated the expression level (RPM) for each replicate separately and compared them to one another. The normalized data of $\log_2$ (RPM value + 1) was used to calculate the correlation coefficient and the correlation between the biological replicates was high (average $R^2 = 0.97$). Dc: the PARE libraries of control group, Dd: the PARE libraries of drought treatment group.

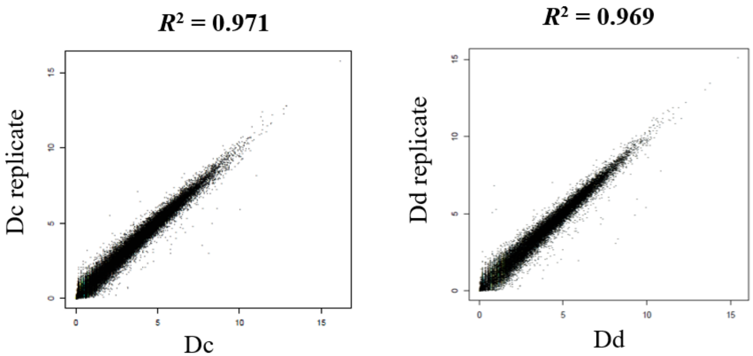

Supplement: Additional file 3. — Correlation between PARE biological replicates. We calculated the expression level (RPM) for each replicate separately and compared them to one another. The normalized data of log2 (RPM value + 1) was used to calculate the correlation coefficient and the correlation between the biological replicates was high (average R 2 = 0.97). Dd: the PARE libraries of drought treatment group, Dc: the PARE libraries of control group. (PDF 65 kb) [file 12870_2015_632_MOESM3_ESM.pdf]
