# Supplementary material for: Global analysis of uncapped mRNA changes under drought stress and microRNA-dependent endonucleolytic cleavages in foxtail millet
Source: BMC Plant Biol. 2015 Oct 6;15:241. doi: 10.1186/s12870-015-0632-0 (PMC4594888; doi:10.1186/s12870-015-0632-0)

Additional file 4: GO functional enrichment analysis for different decay pattern mRNA.

Gene Ontology (GO) analysis was performed for the type I, II, IV genes using WEGO which organizes information for cellular component categories, molecular function and biological process. The Pearson Chi-square test was used for statistical analysis. GO categories that show a significant (α =0.05) enrichment were analyzed and displayed here.

Type I

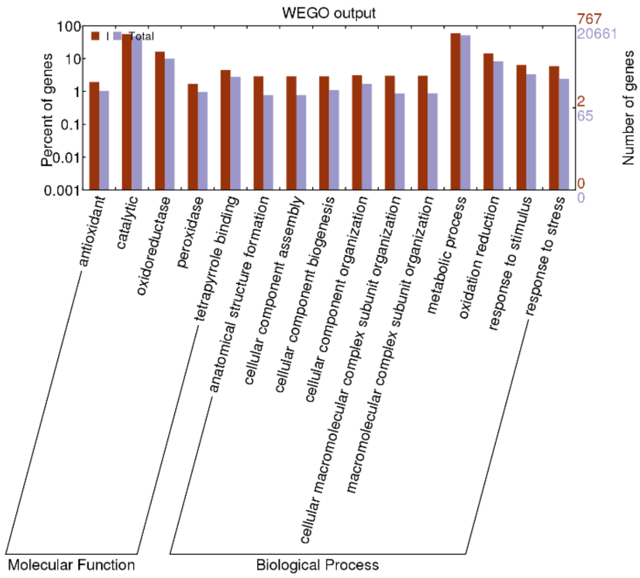

Type II

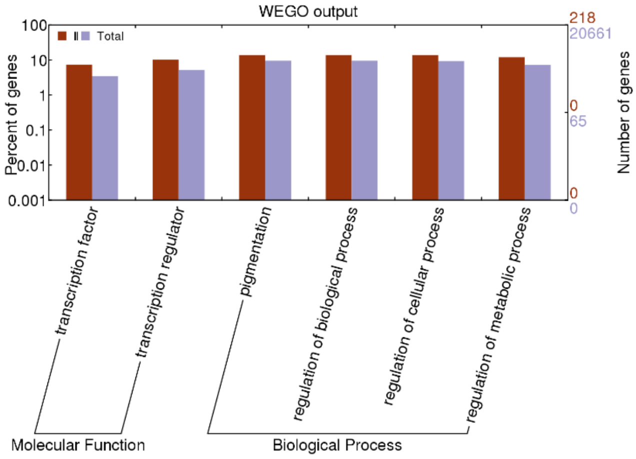

Type IV

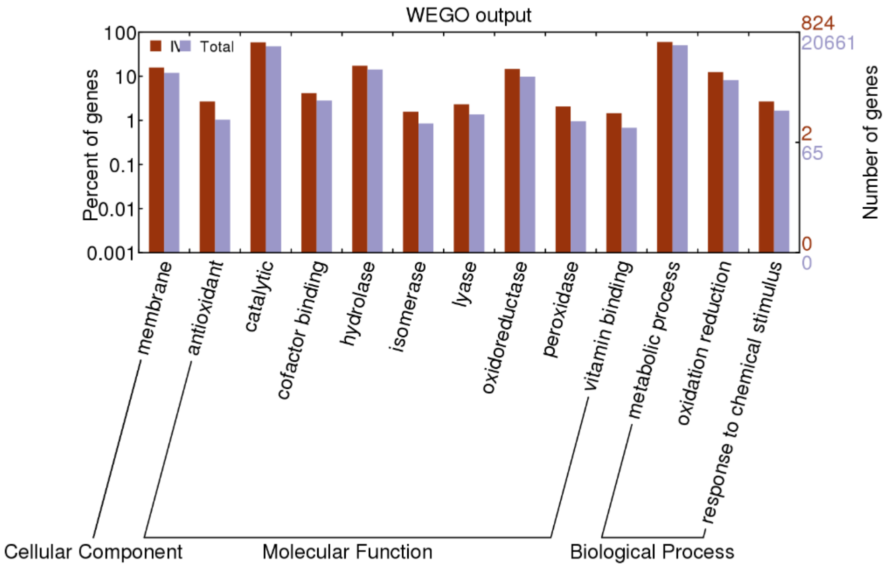

Supplement: Additional file 4. — GO functional enrichment analysis for different decay pattern mRNA. Gene Ontology (GO) analysis was performed for the type I, II, IV genes using WEGO which organizes information for cellular component categories, molecular function and biological process. The Pearson Chi-square test was used for statistical analysis. GO categories that show a significant (α =0.05) enrichment were analyzed and displayed here. (PDF 396 kb) [file 12870_2015_632_MOESM4_ESM.pdf]
