# Supplementary material for: Global analysis of uncapped mRNA changes under drought stress and microRNA-dependent endonucleolytic cleavages in foxtail millet
Source: BMC Plant Biol. 2015 Oct 6;15:241. doi: 10.1186/s12870-015-0632-0 (PMC4594888; doi:10.1186/s12870-015-0632-0)

**Additional file 6: Gene transcript features to different mRNA decay patterns.** Analyzed GC content and MFEI value of mRNA, 5′ UTR and 3′ UTR for type-I, -II , -IV , randomly selected genes and all genes in foxtail millet. “R” represented randomly selected genes, “A” represented all genes, “***” indicate statistically significant differences at *P* value< 0.001 (Student’s wilcox-test).

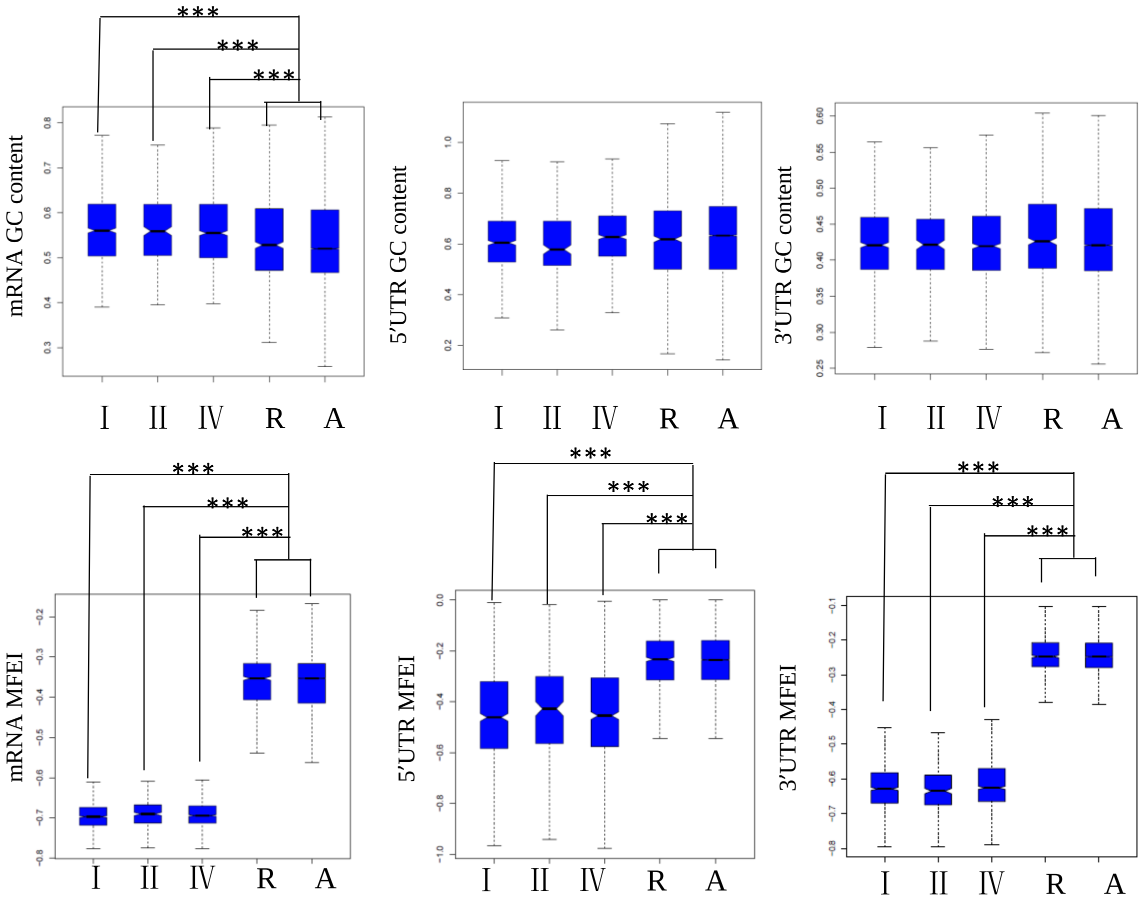

Supplement: Additional file 6. — Gene transcript features to different mRNA decay patterns. Analyzed GC content and MFEI value of mRNA, 5' UTR and 3' UTR for type-I, −II, −IV, randomly selected genes and all genes in foxtail millet. “R” represented randomly selected genes, “A” represented all genes, “***” indicate statistically significant differences at P value < 0.001 (Student’s wilcox-test). (PDF 257 kb) [file 12870_2015_632_MOESM6_ESM.pdf]
