# Supplementary material for: Global analysis of uncapped mRNA changes under drought stress and microRNA-dependent endonucleolytic cleavages in foxtail millet
Source: BMC Plant Biol. 2015 Oct 6;15:241. doi: 10.1186/s12870-015-0632-0 (PMC4594888; doi:10.1186/s12870-015-0632-0)

Additional file 9: Foxtail millet PARE tags matching miRNA hairpins. (A) “Possible loop-last” processing of miRNA hairpins. (B) “Loop-first” processing of miRNA hairpins. (C) Unexpected cleavage signals in several miRNA hairpins. Regions within the pink and blue bars indicate the positions of the miRNA and miRNA* in the precursor, respectively. The read count at each position is indicated as a scatter plot.

A: Loop last

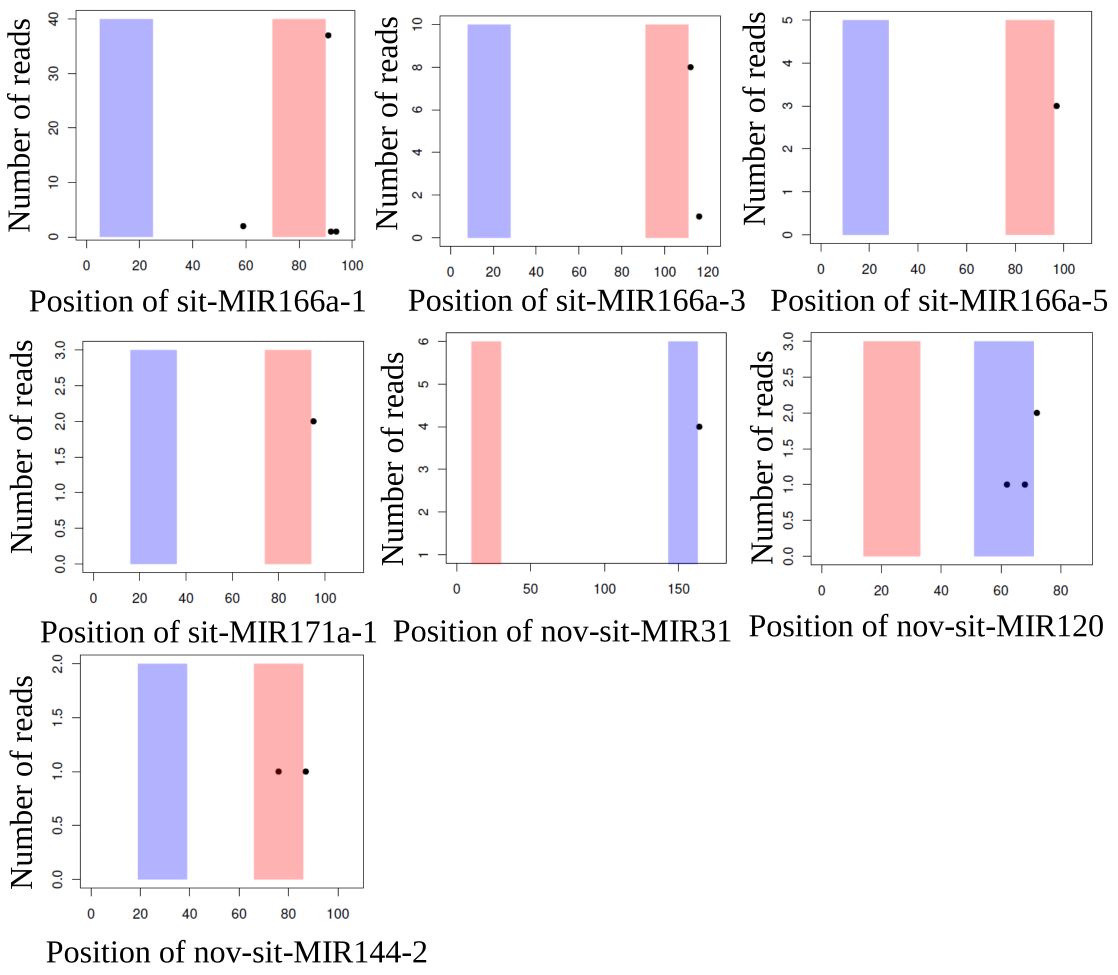

B: Loop first

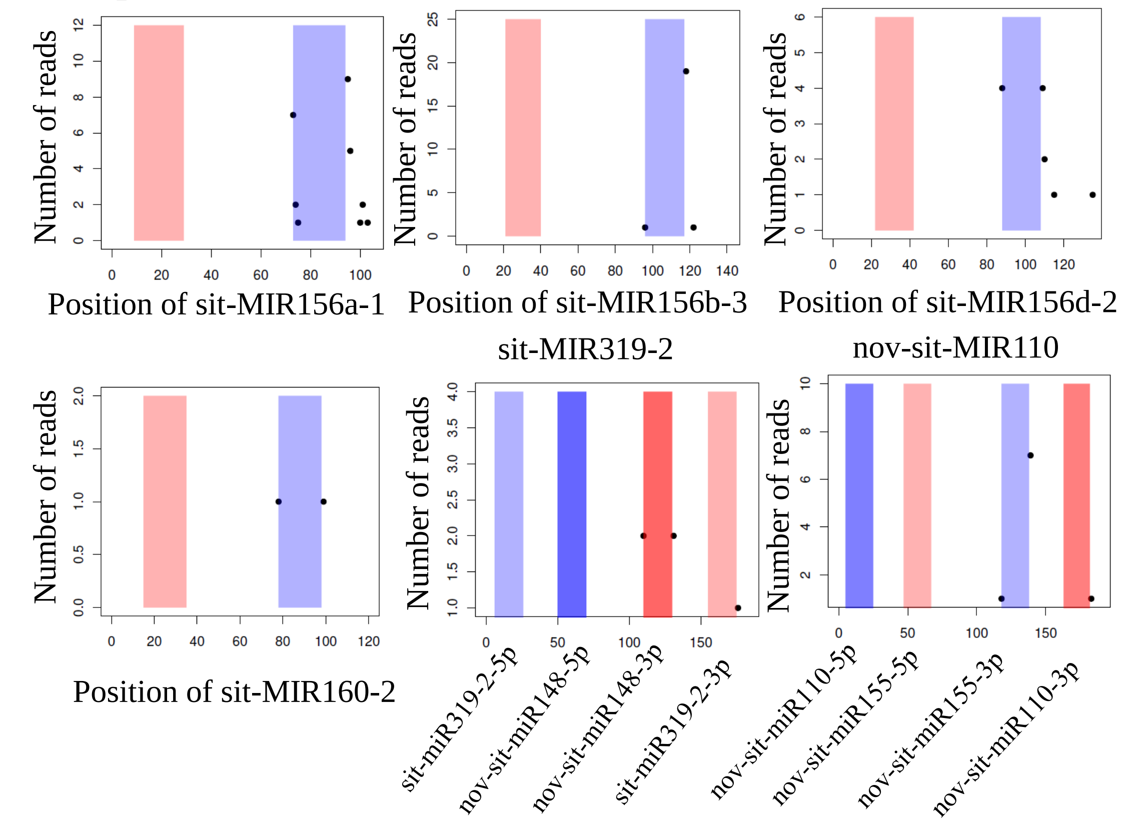

C: Special

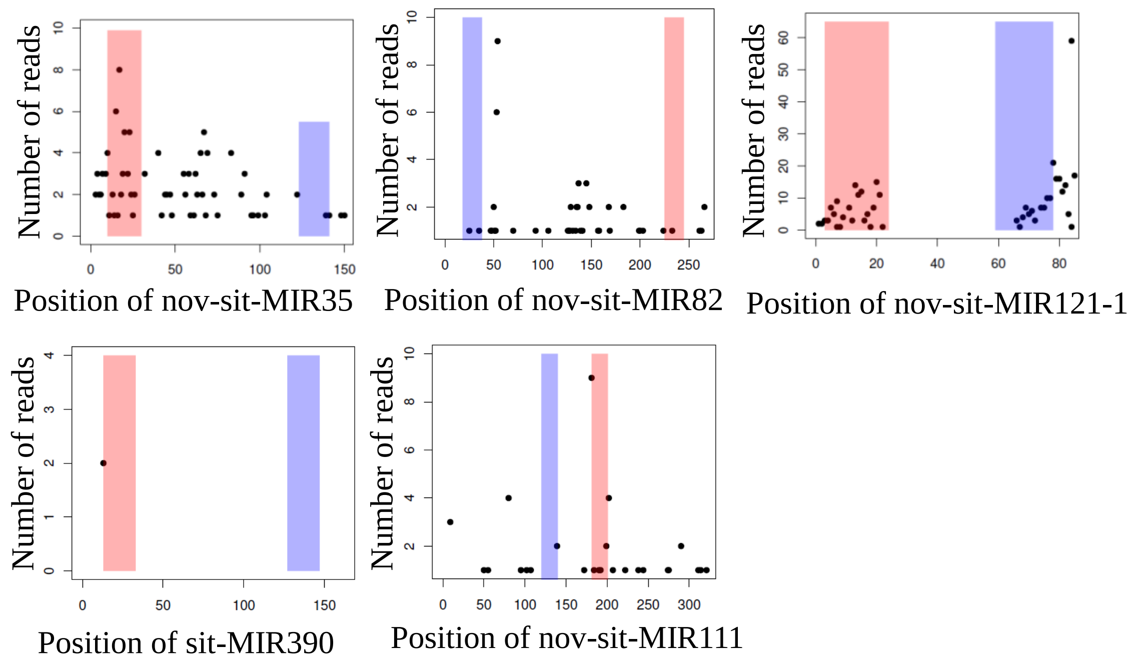

Supplement: Additional file 9. — Foxtail millet PARE tags matching miRNA hairpins. (A) “Possible loop-last” processing of miRNA hairpins. (B) “Loop-first” processing of miRNA hairpins. (C) Unexpected cleavage signals in several miRNA hairpins. Regions within the pink and blue bars indicate the positions of the miRNA and miRNA* in the precursor, respectively. The read count at each position is indicated as a scatter plot. (PDF 356 kb) [file 12870_2015_632_MOESM9_ESM.pdf]
